# Construction and Evaluation of an Artificial Intelligence Assistant Decision-Making System Focused on the Treat-to-Target Framework and Full Process Management for Atopic Dermatitis: Study Protocol for a Randomized Controlled Trial

**DOI:** 10.3390/jcm14093015

**Published:** 2025-04-27

**Authors:** Mengmeng Li, Qingfeng Liu, Yujia Chen, Youqin Liu, Chun He, Jingyi Li

**Affiliations:** Department of Dermatology and Venereology, West China Hospital of Sichuan University, No.37 Guo Xue Lane, Chengdu 610041, China; limengmeng1986@wchscu.cn (M.L.); liuqingfeng@wchscu.cn (Q.L.); 2023224025374@stu.scu.edu.cn (Y.C.); liuyouqing@stu.scu.edu.cn (Y.L.); hechun@wchscu.cn (C.H.)

**Keywords:** atopic dermatitis, treat to target, artificial intelligence assistant decision-making system

## Abstract

**Background/Objectives**: Atopic dermatitis (AD) is a chronic inflammatory skin disease characterized by recurrent rashes and itching, which seriously affects the quality of life of patients and brings a heavy economic burden to society. The treat-to-target (T2T) strategy was proposed to guide optimal use of systemic therapies in patients with moderate to severe AD, and patients’ adherence is emphasized along with combined evaluation from both health providers and patients. While effective treatments for AD are available, non-adherence of treatment is common in clinical practice due to the patients’ unawareness of self-evaluation and lack of concern about the specific follow-up time points in clinics, which leads to the treatment failure and repeated relapse of AD. **Methods**: This project consists of three parts. First, an artificial intelligence (AI) model for diagnosis and severity grading of AD based on deep learning will be trained. Second, an AI assistant decision-making system (AIADMS) in the form of an app will be developed. Third, we design a prospective, randomized controlled trial to test the hypothesis that the AIADMS with implementation of the T2T could help control the disease progression and improve the clinical outcomes. **Results**: A total of 232 participants diagnosed with moderate to severe AD will be included and allocated into the app group or the control group. In the app group, participants will be assisted in using the app during the process of management and follow-up at the scheduled time points, including 2 weeks, 4 weeks, 8 weeks, 12 weeks, 6 months, and 12 months after treatment. In the control group, the diagnosis, treatment, and follow-up of participants will be carried out according to the current routine on a face-to-face basis. The primary outcome is the overall efficiency rate of treating objectives including PP-NRS, EASI, SCORAD, POEM, and DLQI at 12 weeks after treatment, which is calculated as the “Total number of participants with effective treatment of 5 treating objectives/total number of participants *100%”. Spss20.0 software will be used to analyze the data according to the principle of intent to treat. **Trial Registration**: The protocol was registered at the National Institutes of Health Clinical Trials Registry with the trial registration number NCT06362629 on 11 April 2024. **Conclusions**: This study aims to improve AD management by integrating advanced technology, patient engagement, and clinician oversight through AIADMS app to achieve treat-to-target (T2T) goals for effective and safe long-term control.

## 1. Introduction

Atopic dermatitis (AD) is a chronic inflammatory skin disease characterized by recurrent rashes and itching [1]. The prevalence of AD in children is 10–20%, and that in adults is about 1–5% in developed countries. There were 390 million AD patients worldwide in 2019, and the number was estimated to reach 450 million and 520 million by 2024 and 2030, respectively [2]. In 2022, the number of AD patients in China was more than 70 million [3]. For moderate and severe AD, the average course is nearly 10 years, and the overall affected lesion area is more than 30% of the skin. Most patients suffering from AD have difficulty sleeping due to unbearable itching, and reducing itching symptoms is the crucial and urgent need for 75.8% of them. More than 10% of them have suicidal tendencies, and 71.2% of them have experienced discrimination. In addition, more than 75% of dermatologists are not satisfied with the existing treatment plan [4].

AD is primarily driven by the activation of the Th2 immune response, with interleukins (IL)-4 and IL-13 playing central roles in its pathophysiology. Both cytokines are critical in the atopic march and contribute significantly to AD symptoms. IL-4 and IL-13 are encoded by genes on chromosome 5, and their polymorphisms are linked to a genetic predisposition for AD in both children and adults. These cytokines promote Th2 cell differentiation and the production of inflammatory cytokines, which recruit eosinophils, mast cells, and basophils, exacerbating the inflammatory response. IL-4 and IL-13 also contribute to pruritus by stimulating sensory neurons and interacting with the IL-31 pathway. Furthermore, they impair skin barrier function by downregulating epidermal proteins and altering lipid composition, leading to increased transepidermal water loss and susceptibility to infections. IL-13 also plays a unique role in tissue remodeling, promoting collagen production and fibrosis in chronic AD lesions [5].

**Figure 1:**
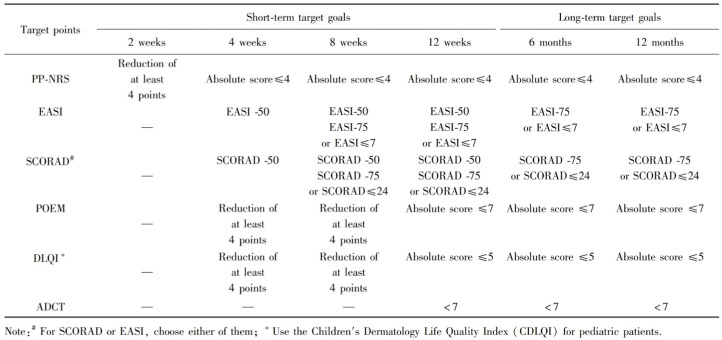
Target threshold at different time points in systemic treatment of moderate to severe atopic dermatitis under the treat-to-target concept [6,7,8].

In August 2022, “Expert Recommendations on Treat-to-Target (T2T) in the Systemic Treatment of Moderate to Severe Atopic Dermatitis” [6] was published, which was combined with the specificities of AD management in clinical practice in China and proposed the treatment objectives and the dynamic adjustment of the treatment plan based on regular follow-up evaluation (Figure 1). The central point of T2T is how to maximize the integration and utilization of relevant resources to help patients achieve persistent control of the disease effectively and safely [7,8]. It is emphasized that the combination of evaluation from both patients and clinicians by applying different evaluative tools, including the Eczema Area and Severity Index (EASI), Patient-oriented Eczema Measure (POEM), Peak Pruritus Numerical Rating Scale (PP-NRS), Scoring Atopic Dermatitis (SCORAD) Index, and Dermatology Life Quality Index (DLQI), which can improve the compliance of patients and provide an important clinical decision-making basis for clinicians [7,8,9]. However, due to the lack of professional medical knowledge and different levels of education, it is difficult for patients to evaluate themselves. Furthermore, clinicians often find it difficult to evaluate patients with all the tools and provide psychological support for patients at clinics because of the limited visiting time. Therefore, the key issues for realization of T2T in AD patients include enhancing the patients’ participation and self-evaluation in systemic treatment, promoting regular follow-up, and effective communication between dermatologists and patients. With the rapid development of mobile technology and artificial intelligence (AI), applications (apps) on smart terminals, including smartphones or pads, with AI assistant decision-making function in the medical field have been explored. Apps can integrate images, documents, videos, and other forms of data and make the communication between doctors and patients effective and convenient, regardless of time and place restrictions. Previous studies have shown that app auxiliary management for diabetes and asthma increase the self-management and participation of patients, significantly promote patients’ compliance, and improve clinical outcomes and prognosis, and 80% of patients believe that this kind of service is a feasible alternative to face-to-face treatment [10,11]. Benefiting from AI deep learning, AD could be diagnosed and evaluated with the utilization of convolutional neural networks (CNNs) models. In a CNN model trained from 4740 clinical images based on the EfficientNet-b4 architecture, AD could be diagnosed with an accuracy of 92.57%, sensitivity of 94.56%, and specificity of 94.41% by the AI dermatology diagnosis assistant in the form of a smartphone-based platform [12]. The severity of AD could be measured objectively after CNN model training by the Inception ResNet-v2 algorithm from a dataset including 9192 cropped AD images [13]. Moreover, SCORAD can be calculated automatically with the application of the Resnet-34 architecture for lesion surface segmentation and the EfficientNet-B0 network architecture for visual sign severity grading [14]. Moreover, recent studies have emphasized the importance of AI in dermatology and other medical fields, further highlighting the potential of AI technologies. Li et al. reviewed the current developments and future trends of AI in dermatological image analysis, identifying key areas where AI could enhance clinical decision-making, particularly in lesion detection and classification [15]. Similarly, Hogarty et al. discussed the broader applications of AI in dermatology and its future directions, noting that AI has become an essential tool in advancing diagnostic precision and personalized treatments [16]. These studies further underscore the relevance of integrating AI into AD management for better patient outcomes. Based on the literature, AI assistant management for AD could be realized in clinical practice to increase diagnostic accuracy and save time for both clinicians and patients. However, to the best of our knowledge, no platform integrating AI deep learning and interactive communication with patients focused on the whole process of AD management including diagnosis, severity evaluation, therapeutic goals, and follow-up has been developed.

In this project, we plan to develop an artificial intelligence assistant decision-making system (AIADMS) in the form of an app based on AI deep learning from massive clinical images of AD target lesions, with implementation of the T2T framework and full follow-up for AD patients. We hypothesize that the goal of the T2T could be better achieved by application of the AIADMS for AD patients, and a clinical randomized controlled trial is designed to verify this assumption.

## 2. Materials and Methods

### 2.1. Automatic Detection and Evaluation of AD Based on AI Deep Learning

#### 2.1.1. Dataset of Atopic Dermatitis

The dataset will be established from more than 10,000 clinical images of AD patients for AI deep learning. Low-quality images, those with resolutions less than 640 × 480 pixel or too unclear to identify the structure of the skin lesions, will be excluded. The images containing the surrounding background will be treated by labeling the border of the body or the limb and cropping the labeled part from the image to include only the AD lesions on the skin, and they will be standardized by an image tagging tool to ensure consistency across the dataset.

#### 2.1.2. Labeling the Clinical Signs of Skin Lesions

The labeling will be completed by three certified dermatologists and three trained algorithm engineers. The dermatologists will label the clinical signs, including erythema, papulation, edema, oozing, excoriation, lichenification, and dryness, and the severity of each sign will be evaluated and labeled on a four-point scale (0: none, 1: mild, 2: moderate, and 3: severe). The result of each clinical sign in an image will be labeled as an example of erythema-2, edema-2, or oozing-3. After labeling the images, the dermatologists and algorithm engineers will verify the quality of the labeled images based on both clinical and labeling rules and cross-validate the accuracy of signs and severity. Images that meet the requirements will be used for model training. During the labeling and model training process, the relevant personnel will be unaware of all the private patient information.

#### 2.1.3. Model Training

The model training will be carried out after labeling of the images. An accurate and efficient semantic segmentation model will be trained to distinguish abnormal skin lesion areas to identify all the clinical signs. A fast and accurate pixel-level skin segmentation model will be trained to determine the ratio of the lesion area to the overall skin area. In addition, an efficient and practical method to convert the segmented skin lesion area into real skin area units will be created to achieve the accurate restoration of the true size as much as possible from the distortion of the skin lesion due to the shooting distance, angle, or automatic enhancement. The dataset will be divided for training, validation, and testing. Images of 6500 of the 10,000 will be used in training and validation of the proposed model, and images of the remaining 3500 of the 10,000 will be used for testing. After training, combined with the different questionnaire items filled out by patients, the evaluative tools including EASI, SCORED, POEM, pp-NRS, and DLQI will be calculated by the model.

### 2.2. Development of the AIADMS App

**Figure 2:**
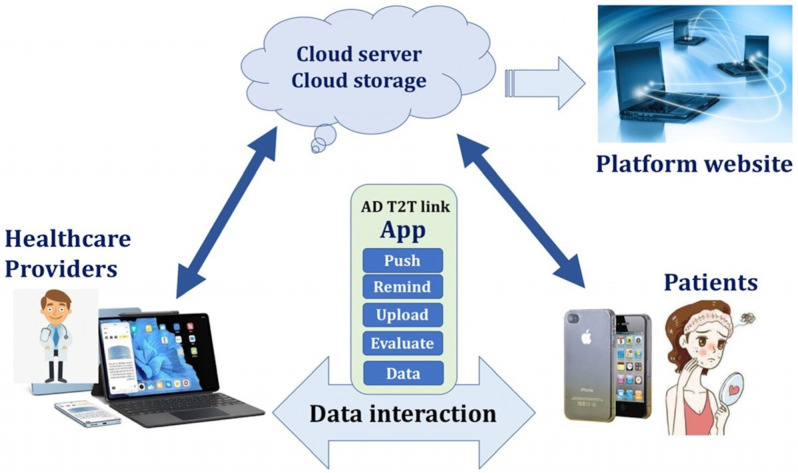
Design concept and fundamental function of artificial intelligence assistant decision-making system (AIADMS) in the form of an app and data interaction mode between healthcare providers and patients.

The app will support the Android system and the IOS system, and it will be designed as two versions for both patients and clinicians with the distinguished login entrance. The fundamental functions of the app will include “Push”, “Reminder”, “Upload”, “Evaluation”, and “Data management” (Figure 2 and Supplemental Figure S1).

The “Push” function is designed to transmit information to patients and medical staff. The pushed information could be received and displayed on the screen of the mobile phone even if the app is not opened and the mobile phone is in the locked screen state, and the users can set the time of receiving the pushed information by themselves. For example, the predetermined time point for follow-up in clinics will be presented as “You should come to see the doctor on next Monday, 25 July 2023.” The “Push” function can activate the use of the app, increase the viscosity of users, and drive the utilization of other functional modules.

The “Reminder” function is mainly used for reminding the patients about taking medicine, uploading photos of skin lesions, self-evaluation, and scheduled follow-up.

The “Upload” function is designed to help patients participate in the systemic treatment. They can upload their photos of skin lesions, the description of progresses of AD, or questionnaires.

The “Evaluation” function is developed to provide information for both patients and medical staff. By uploading photos of skin lesions and filling in the different questionnaire items, the app will automatically evaluate the severity of lesions and calculate the EASI, POEM, PP-NRS, SCORAD, or DLQI scores. This function could help patients know more about their situation related to the disease and take part in self-evaluation and self-care as the T2T strategy recommends.

The “Data management” function is designed for medical staff to manage the patients more conveniently and design the medical research. They can log in to the app platform website to collect and export data and carry out statistical analysis and big data mining. The app itself can also do simple statistics and management of data. For example, data such as EASI, POEM, and PP-NRS scores at the time points of before treatment, 2 weeks, 4 weeks, 12 weeks, and 6 months after treatment could be automatically generated into statistical reports to be presented in the form of histograms or curves. The app can also be further improved and updated to the new version through the analysis of users’ habits, and the function modules could be optimized with the high frequency of use and the feedback from both medical staff and patients.

### 2.3. Study Design

**Figure 3:**
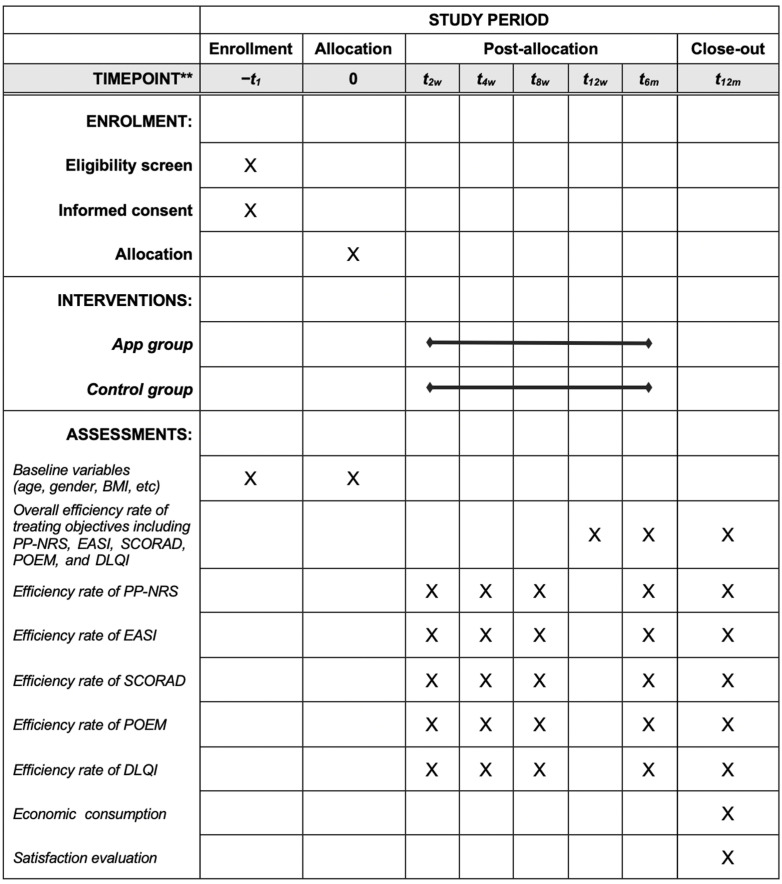
SPIRIT schedule of enrollment, interventions, and assessments. ** List specific timepoints in this row.

This is a single-centered, prospective, randomized controlled trial that tests the superiority of the implementation of a T2T strategy by application of an AIADMS app in patients with AD in terms of improvement of clinical outcomes. It follows the CONSORT statement (http://www.consort-statement.org/ accessed on 24 February 2025) and is conducted under the regulations of the Declaration of Helsinki. A flow diagram of this study is demonstrated in Figure 3.

#### 2.3.1. Participants

Inclusion criteria: patients diagnosed with moderate to severe AD, aged 2–75 years; be able to communicate in Chinese; with basic reading and writing skills; participants or the guardian have smartphones or pads and are familiar with use skills.

Exclusion criteria: mental illness; personality disorder; language barrier; hearing impairment; communication difficulties; with serious coexisting diseases, such as cardiopulmonary insufficiency, liver dysfunction, renal dysfunction, blood system diseases, tumors, or other diseases; other situations that are not suitable for participating in the clinical trial.

#### 2.3.2. Randomization and Blinding

According to the sequence of time wherein the participants are enrolled, after entering the screen number and the individual’s information, the computer-based randomized number prepared on the Internet and the allocated group can be retrieved on the website of the CRS. While the statisticians responsible for final analysis will be blinded to the treatment assignment, the participants, investigators, healthcare providers, research assistants, and the responsible physicians will not be blinded.

#### 2.3.3. Intervention

Participants will be allocated to the “App group” or the “Control group”.

In the app group, participants will be assisted in using the app during the process of management and follow-up at the scheduled time points, including 2 weeks, 4 weeks, 8 weeks, 12 weeks, 6 months, and 12 months after treatment, and the evaluation of five treating objectives including PP-NRS, EASI, SCORAD, POEM, and DLQI should be done on the day of follow-up.

In the control group, the diagnosis, treatment, and follow-up of participants will be carried out according to the current routine on a face-to-face basis. The time points of the participant follow-ups will be determined by the responsible dermatologist, and the evaluation of five treating objectives including PP-NRS, EASI, SCORAD, POEM, and DLQI will be done and recorded on the day of follow-up.

#### 2.3.4. Outcomes

Primary outcome:

The primary outcome is the overall efficiency rate of treating objectives including PP-NRS, EASI, SCORAD, POEM, and DLQI at 12 weeks after treatment, which is calculated as “Total number of participants with effective treatment of 5 treating objectives/total number of participants *100%”. Criteria for effective treatment are as below: ① PP-NRS absolute score ≤ 4; ② EASI-50, EASI-75, or EASI ≤ 7; ③ SCORAD-50, SCORAD-75, or SCORAD ≤ 24; ④ POEM absolute score ≤ 7; ⑤ DLQI absolute score ≤ 5.

Secondary outcomes:

- The respective efficiency rate of PP-NRS, EASI, SCORAD, POEM, and DLQI, which is calculated as “Total number of participants with effective treatment of PP-NRS, or EASI, or SCORAD, or POEM, or DLQI/total number of participants *100%”;
- The economic consumption such as the travel expenses, expenses with lost working days, accommodation expenses, medical expenses, etc.;
- Satisfaction evaluation, defined as the assessment for the overall medical services by answering questions about their level of satisfaction with healthcare: 0 means completely dissatisfied, and 100 means very satisfied, and the participants or their guardians give a score between 0 and 100.

#### 2.3.5. Sample Size Calculation

Based on our preliminary investigative results, we estimate that the overall efficiency rate is 35% (unpublished data), and we hypothesize that the overall efficiency rate increases to 55%, which is clinically significant, after the implementation of the app. Assuming the difference between the two groups was a 5% significance level and a power of 0.80, 96 participants in each group are required. Considering an estimated 20% dropout rate, 116 participants in each group and a total of 232 participants are required in this trial.

#### 2.3.6. Study Timeline

**Figure 4:**
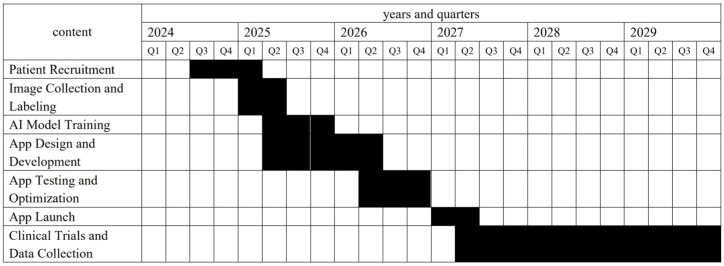
Study timeline and key milestones. The timeline outlines the major phases of the study from 2024 to 2029, including patient recruitment, image collection and labeling, AI model training, app design and development, app testing and optimization, app launch, and clinical trials and data collection. Each phase is represented across the respective quarters to illustrate the sequential progression and overlap of tasks throughout the study period.

The study timeline is illustrated in Figure 4, which outlines the key milestones and phases of the study, from patient recruitment to data analysis.

#### 2.3.7. Statistical Analysis

Spss29.0 software will be used to analyze the data according to the principle of intent to treat. Demographic data are described by mean, standard deviation, frequency, median, percentage, etc. All data are tested for normal distribution, the *t*-test is used for continuous variables that conform to normal distribution, the chi-squared test is used for classified variables, and a nonparametric test is used for variables that do not conform to normal distribution. After 2, 4, 8, and 12 weeks of treatment, the sub item compliance rates of PP-NRS, EASI, SCORAD, POEM, and DLQI are analyzed by repeated measures of variance. *p* < 0.05 means that the difference is statistically significant.

### 2.4. Ethics and Dissemination

The protocol (Version 1.0) was approved by the Biological- Medical Ethical Committee of the West China Hospital of Sichuan University with approval No. 2023(2443) on 1 February 2024.

Written informed consent (see Supplementary File) will be obtained from all participants before we take images of the skin lesions and before we recruit them in clinical trials. The results of the project will be reported in a peer-reviewed journal.

### 2.5. Ethical Considerations and Data Privacy

This study complies with ethical guidelines for clinical research and data protection. All personal information, including clinical images, will be anonymized, with patient identifiers replaced by coded data. Only authorized personnel will have access to the linkage between codes and patient identities, stored separately in a secure database.

Sensitive clinical images will be stored on encrypted servers with restricted access, and data transmission will use encryption protocols to prevent unauthorized access. To minimize biases in AI-assisted decision-making, the algorithms will be trained on diverse datasets and monitored regularly for fairness and accuracy. An independent ethics committee will oversee any ethical issues related to AI use in clinical care.

Participants will be fully informed about data usage and their right to withdraw at any time. The study has received approval from the institutional ethics committee and complies with all applicable privacy regulations.

## 3. Discussion

We investigated the current situation of the AD patients and dermatologists in our clinic, recorded the visiting time for AD patients in our clinic, and found that if we evaluated a patient only with EASI, it would take an experienced dermatologist about 20 min. If we evaluated the patient with all the tools, including EASI, POEM, SCORAD, PP-NRS, DLQI, and ADCT, to see whether the T2T goal was achieved, at least 90 min would be needed. However, only 5 to 10 min could be allocated for each patient in clinic, and because there would be about 45 to 60 patients in 4 h of an outpatient visiting clinic, the patient’s medical and psychological needs would be far from met. Besides, due to the lack of knowledge of the disease, some of the patients use different medications randomly with eagerness for the rapid disappearance of the skin lesion or discontinue therapy when they feel that the symptoms have improved even a little bit without consultation with the doctor, and most of the AD patients have no idea about self-participation and clinician-patient combined management of AD, which make it difficult to control the disease or improve the clinical outcomes. It is crucial for both clinicians and AD patients to develop a convenient way to save time and have continuous management.

For the primary outcome, all five items including PP-NRS, EASI, SCORAD, POEM, and DLQI need to show improvement to meet the criteria for effective treatment as the primary outcome, and the goal of T2T could be regarded as achieved. These measures encompass both objective and subjective therapeutic effects, ensuring a comprehensive assessment of treatment efficacy. Improvement in each of these parameters is required to consider the treatment effective, with each criterion contributing essential information to the overall evaluation of treatment success.

This is an interdisciplinary project combining theories of clinical dermatology, computer science, and psychology and focusing on implementing a T2T framework for AD. Development of this app will be focused on patients’ participation in treatment, combination of evaluation from both patients and clinicians by applying different evaluation tools at different time points, improving the compliance of patients, and providing important an clinical decision-making basis for clinicians in order to maximize the integration and utilization of relevant resources to help patients achieve persistent control of the disease effectively and safely. According to “Expert Recommendations on Treat-to-Target (T2T) in the Systemic Treatment of Moderate to Severe Atopic Dermatitis” [6] published in August 2022 in China, the follow-up assessments are set at the time points of 2 weeks, 4 weeks, 8 weeks, and 12 weeks after treatment for evaluation of the short-term target goals and 6 months and 12 months after treatment to evaluate the long-term target goals (Figure 1). With the combinative assessment from both the dermatologists and the patients assisted by the app, the clinicians could determine whether the goals of T2T are met at the follow-up and how to proceed with the following treatment to achieve persistent control of AD.

The reliability of AI in evaluating cutaneous lesions remains a topic of debate, particularly in real-world clinical practice, where factors such as image resolution and the presence of artifacts can impact its performance. To mitigate these challenges, we plan to conduct rigorous external validation studies using a diverse set of images from various clinical settings, ensuring the robustness and generalizability of the AI model. These validation efforts will be complemented by expert reviews from independent dermatologists to confirm the system’s clinical relevance. Additionally, the AI system has been specifically designed to address cases where multiple dermatoses coexist in the same anatomical region, such as atopic dermatitis and acne on the face. Through advanced segmentation techniques and a multi-class classification approach, the system is capable of differentiating and accurately diagnosing individual lesions, even in complex presentations with overlapping skin conditions. This capability will be critical for ensuring accurate diagnoses and effective treatment recommendations in patients with multiple concurrent dermatological issues.

Since AD is a chronic disease, a key challenge in managing it through mobile application is ensuring long-term user engagement, and prolonged app use is important to improve adherence. To address this, the app will incorporate several strategies aimed at promoting sustained use. Personalization is central, with the app adapting content to the individual’s treatment progress and providing continuous feedback that encourages adherence. Additionally, gamification features, such as reminders, rewards, and goal tracking, are included to enhance user motivation and engagement. The app’s design is intuitive and user-friendly, specifically tailored to reduce cognitive load for users while maintaining ease of navigation. Periodic updates to the app, along with adaptive learning algorithms, ensure that the content remains relevant and responsive to evolving user needs. Moreover, the integration of support systems, including caregiver involvement and community engagement, provides a social support network that further encourages ongoing app use. These multifaceted strategies aim to create a sustainable, supportive environment that fosters long-term adherence, which is critical in the management of chronic conditions like AD.

The key strength of this study lies in the development of an innovative mobile application aimed at addressing limitations in the current management of atopic dermatitis (AD), particularly the time constraints in clinical settings and the lack of patient involvement. By enabling more comprehensive and efficient assessments, the AIADMS app has the potential to enhance treatment adherence and improve clinical outcomes. The app’s ability to streamline clinical workflows and provide real-time feedback empowers both clinicians and patients, fostering a more proactive approach to disease management.

However, several limitations should be acknowledged. First, the AIADMS app may face technical challenges, including issues related to accuracy, integration with existing clinical workflows, and potential difficulties with the user interface. These factors could affect both the usability and effectiveness of the app. Second, variability in patient adherence may influence study outcomes, as inconsistent use of the app might result in suboptimal disease management. Third, the study is conducted in a single center in China, which may limit its applicability to other populations with different healthcare systems and patient demographic or clinical characteristics, and generalizability of the results to other populations remains uncertain, given that the study focuses on a specific patient group. Further multi-centered studies involving diverse populations will be required to confirm the broader applicability of the AIADMS app. Additionally, a limitation of this study is the open-label, blinded end-point design, which makes it impossible to mask the assignment of participants. Both the participants and the investigators will be aware of group allocations, potentially introducing bias, particularly as participants in the AI-assisted group may have expectations of improved outcomes, which could influence self-reported measures, such as pruritus levels or the DLQI. To address this, the potential impact of the lack of blinding is being mitigated by having each participant’s condition evaluated by three clinicians to achieve a comprehensive assessment. Both groups will receive comparable levels of attention and care throughout the study, ensuring consistency in participant engagement. Additionally, objective outcome measures, including clinician-assessed severity of symptoms, will be incorporated to supplement self-reported data and provide a more comprehensive evaluation of treatment effects. Furthermore, all participants will be assigned unique identification numbers, with their individual data registered under these numbers. These data will be transferred to data collectors and statisticians who remain blinded to the group assignments, thus minimizing the risk of statistical bias during data analysis.

The study also relies on objective outcome measures like the Eczema Area and Severity Index (EASI), which offers standardized and quantifiable assessments of disease severity, reducing the influence of subjective judgment. Strict adherence to standardized treatment protocols and follow-up assessments across all groups will ensure consistency in care and data collection, minimizing variability and enhancing the reliability of the findings.

## 4. Conclusions

In conclusion, despite the challenges posed by time constraints in clinical settings and the lack of full blinding, this study aims to improve the management of AD through the integration of advanced technology, patient engagement, and clinician oversight. By focusing on achieving treat-to-target (T2T) goals, we believe that the AIADMS app has the potential to facilitate more effective and safe long-term control of AD.

## Data Availability

The datasets are not readily available currently. Individual participant data with anonymity will be uploaded to the IPD sharing platform to achieve data sharing. Data will be available after the agreement of the correspondence author upon reasonable request.

## Supplementary Materials

The following supporting information can be downloaded at https://www.mdpi.com/article/10.3390/jcm14093015/s1: Figure S1: Demonstration of fundamental function of artificial intelligence assistant decision-making system (AIADMS) in the form of an app.

## Abbreviations

The following abbreviations are used in this manuscript:

AD	atopic dermatitis
AI	artificial intelligence
AIADMS	AI assistant decision-making system
T2T	treat to target
QoL	quality of life
EASI	Eczema Area and Severity Index
POEM	Patient-oriented Eczema Measure
PP-NRS	Peak Pruritus Numerical Rating Scale
SCORAD	Scoring Atopic Dermatitis
DLQI	Dermatology Life Quality Index
