# Supplementary figures and images for: Construction and Evaluation of an Artificial Intelligence Assistant Decision-Making System Focused on the Treat-to-Target Framework and Full Process Management for Atopic Dermatitis: Study Protocol for a Randomized Controlled Trial
Source: J Clin Med. 2025 Apr 27;14(9):3015. doi: 10.3390/jcm14093015 (PMC12072663; doi:10.3390/jcm14093015)

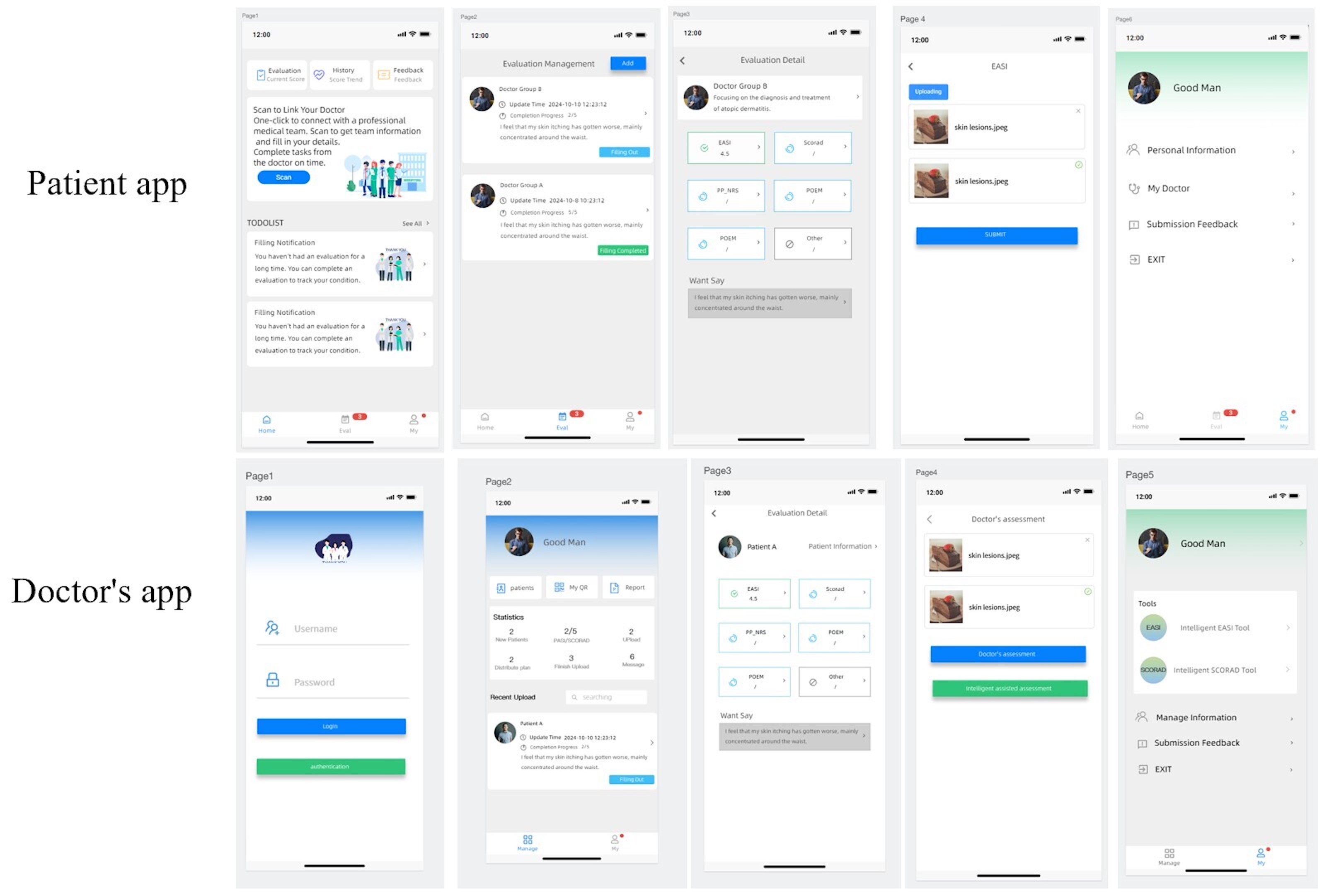

Supplement: Supplementary file 1 [file jcm-14-03015-s001.zip › jcm-3494480-supplementary.jpg]
